# An Energy-Efficient Algorithm for Wearable Electrocardiogram Signal Processing in Ubiquitous Healthcare Applications

**DOI:** 10.3390/s18030923

**Published:** 2018-03-20

**Authors:** Ali Hassan Sodhro, Arun Kumar Sangaiah, Gul Hassan Sodhro, Sonia Lohano, Sandeep Pirbhulal

**Affiliations:** 1 Electrical Engineering Department, Sukkur IBA University, Sukkur 65200, Pakistan; 2 Decision Information Systems and Production LAB, University Lumiere Lyon2, Bron-69500, France; 3 School of Computing Science and Engineering, VIT University, Vellore 632014, Tamil Nadu, India; arunkumarsangaiah@gmail.com; 4 Department of Physics, Shah Abdul Latif University, Khairpur Mirs 66111, Sindh, Pakistan; hassangull183@gmail.com; 5 Department of English, University of Sindh, Jamshoro 71000, Sindh, Pakistan; sonialohano1995@yahoo.com; 6 Shenzhen Institutes of Advanced Technology, Chinese Academy of Sciences, Shenzhen 518055, China

**Keywords:** electrocardiogram (ECG) signal processing, wearable platform, noise, energy-efficiency, Filters, healthcare, ETPC, ubiquitous, beats per minute (BPM)

## Abstract

Rapid progress and emerging trends in miniaturized medical devices have enabled the un-obtrusive monitoring of physiological signals and daily activities of everyone’s life in a prominent and pervasive manner. Due to the power-constrained nature of conventional wearable sensor devices during ubiquitous sensing (US), energy-efficiency has become one of the highly demanding and debatable issues in healthcare. This paper develops a single chip-based wearable wireless electrocardiogram (ECG) monitoring system by adopting analog front end (AFE) chip model ADS1292R from Texas Instruments. The developed chip collects real-time ECG data with two adopted channels for continuous monitoring of human heart activity. Then, these two channels and the AFE are built into a right leg drive right leg drive (RLD) driver circuit with lead-off detection and medical graded test signal. Human ECG data was collected at 60 beats per minute (BPM) to 120 BPM with 60 Hz noise and considered throughout the experimental set-up. Moreover, notch filter (cutoff frequency 60 Hz), high-pass filter (cutoff frequency 0.67 Hz), and low-pass filter (cutoff frequency 100 Hz) with cut-off frequencies of 60 Hz, 0.67 Hz, and 100 Hz, respectively, were designed with bilinear transformation for rectifying the power-line noise and artifacts while extracting real-time ECG signals. Finally, a transmission power control-based energy-efficient (ETPC) algorithm is proposed, implemented on the hardware and then compared with the several conventional TPC methods. Experimental results reveal that our developed chip collects real-time ECG data efficiently, and the proposed ETPC algorithm achieves higher energy savings of 35.5% with a slightly larger packet loss ratio (PLR) as compared to conventional TPC (e.g., constant TPC, Gao’s, and Xiao’s methods).

## 1. Introduction

Internet of things (IoT) and ubiquitous sensing (US) are playing an important role in smart healthcare, and have entirely changed the landscape of the conventional traits and practices with self-organizing, distributed, low-power, and economical features. One of the challenging factors affecting the design and development of US is the energy drain and confined battery lifetime of sensor-enabled devices. A rapid technological revolution in miniaturized wearable devices and fast mathematical tools have motivated every corner of the medical domain, and has encouraged body sensor networks (BSNs) for examining the patients’ health over 24-h. A BSN is a group of tiny sensor nodes deployed on/in-body for getting up-to-date and accurate medical information by consulting with expert physicians for present and future health records. Consequently, the data acquired from the multiple sensor nodes (e.g., electrocardiogram (ECG), blood pressure (BP), saturation oxygen level (SpO_2_), etc.) are further processed, and communicated by transmitter sensor nodes and base station (BS) to the end user. At present, the continuous, dynamic, and long-term acquisition and monitoring of human physiological signals is carried out by wearable smart healthcare devices. The main challenge for conventional portable devices is their high-power consumption, and hence shorter battery lifetime. To remedy these problems, this research develops a wearable single chip-based ECG system for collecting real-time data of heart-rate activity. Because monitoring heart activities (i.e., ECG) patterns with the identification of abnormalities is an important clinical task (particularly for old-age heart-attack patients), ECG is a widely accepted diagnosis tool in clinical validations of heart-related diseases. The impact of respiration-oriented heart functionality on the ECG was first interpreted by Liu et al. [1] and others [2,3]. Due to high maturity of ECG vital signals in the medical industry, human respiratory activity can be extracted from them.

A remarkable revolution in wearable/portable devices has significantly changed the scenario of the medical industry. BSNs are one of the innovative smart healthcare technologies which encourage energy-efficiency in every corner of the medical world. Even from a technical view-point, energy-efficient, reliable, and noise-free communication is the dire need of today’s challenging healthcare markets. Dynamic transmission power control-based approaches are potential and possible remedies for offering longer and power-efficient operation, with longer battery lifetime in BSNs. In the mean-time, due to increased performance and lower prices, medical media is everywhere, and mostly all personal computers and mobile devices are capable of handling medical multimedia content. Additionally, in recent years, the convergence of the digital media in medical health and the information communications technology (ICT) industries has become the center of attention. So, high power drain and shorter battery lifetime are considered as critical challenges. For example, if a physician wants to rapidly check an ECG report of an emergency elderly patient for providing quick and timely treatment, noise-free and more visible data are required, which can be obtained by power-efficient, reliable, and longer battery lifetime wearable devices. Several ECG-oriented strategies have already been designed, developed, and discussed for examining and evaluating different diseases—for instance, a belt-based method which is badly affected by random errors and noise while extracting the results. It is evident and has been proved by many previous studies that human heart-rate activity is impacted heavily by respiration patterns and its extraction from other derived components [4]. The ECG-supported respiration environment is simple, economical, and easy to handle in medical hospitals and theatres for long-lasting check-up and diagnosis of patients with several leads (i.e., channels to adapt various patients at a time) [5,6,7]. As typical wearable devices are based on photoplethysmography (PPG), they are larger in size and not easy to attach on the body of the patients while walking, running, or exercising. Besides, typical traditional devices lack continuous monitoring of activity/data. The main significance of this research is to help physicians/medical doctors to monitor their patients remotely with energy-efficient wearable devices by collecting any visible symptoms/signs that may occur during exercise or daily-life activities. The best application of the wearable ECG is for old-age patients while performing sport activities. Besides, the developed energy-efficient wearable ECG chip will help trainers to control the exercise load of their trainees. This developed chip will monitor the heart and respiration rates; in case their value exceeds the specified threshold, then the trainer will stop exercise immediately. However, here we will only focus on heart-rate activity monitoring (i.e., ECG).

One of the most challenging problems in digital signal processing (DSP) is to receive the information signal without any loss. It is important to reduce the attenuation generated by random noise to improve the performance of the desired signal. As we know, digital signal is not a natural phenomenon; it is generated from its analog counterpart. The analog signal is uniformly sampled at a sampling rate/Nyquist rate $fs=2\times f_{\max}$, and after that sampled signal is quantized and encoded to get the final digital signal. When the signal transmits through the channel, it faces some random disturbances due to environment or analog-to-digital conversion processes, which is normally distributed at the time termed as additive white Gaussian noise (AWGN). This noise adds to the original signal and produces errors and noise, and hence quality will be degraded. Therefore, it can be said that the received signals are a mixture of information and noise. One of the main tasks of this research is to extract the desired information from the original signal, which is a very cumbersome task; also, statistics of the noise corrupting a signal are unknown in many situations and change with time. Moreover, the power of noise may be greater than the power of the desired signal being transmitted [8]. Due to noise, more power will be consumed during communication, and major parts of the data transmission are done through smart wearable mobile devices, which consume more power and have shorter battery lifespans; thus, power-efficient and accurate techniques are needed for tiny sensor nodes. Emerging medical markets of wearable devices have increasingly added to their value and importance, even in non-medical activities such as sports and business, so energy-efficiency is a dire need for continuous diagnosis and management of bio-signals in the smart healthcare environment. Authors in [9,10,11] developed a hardware platform with wearable devices for various medical health applications; similarly, researchers in [12,13] propose the standards for telecardiology, telemedicine, and medical portable devices according to the application requirements.

Most of the aforementioned studies were conducted using separate transmission power control algorithms for individual tasks (e.g., vital signal monitoring, clinical diagnosis on the basis of heart-rate activity, power management in wireless sensor network (WSN)/WBSN merely adopting software or hardware at a time. However, no-one focused on the specific wearable device development, quantification, and examination of real-time ECG data, the development of an energy-efficient algorithm and its validation through an integrated hardware and software platform. To the authors’ knowledge, this is the beauty of our research which differentiates it from previous work.

The major contributions of this paper are three-fold. First, to develop a single chip-based wearable electrocardiogram (ECG) with pervasive or ubiquitous sensing features by adopting an analog front end (AFE) chip model ADS1292R. Second, to design notch, low-pass, and high-pass filters and implement these on the hardware for further rectifying the power-line noise and artifacts in real-time ECG data. Third, to propose a transmission power control (TPC)-based energy-efficient (ETPC) algorithm for power optimization during pervasive sensing and transmission achieving energy savings of up to 35.5%, then to implement and validate by considering human ECG data ranging from 60 beats per minute (BPM) to 120 BPM with 60 Hz noise on the joint hardware and software platform and compare with conventional TPC methods.

The rest of the paper is structured as follows. Section 2 reviews the rigorous literature about ECG monitoring and filtering methods. Section 3 discusses the methods and materials, including filter design. The energy-efficient TPC-based ECG transmission algorithm is proposed in Section 4. Experimental results are examined in Section 5, and the paper is concluded in Section 6.

## 2. Related Work

Several researchers have discussed ECG monitoring and noise reduction for the longer life-expectancy of patients; some of the relevant studies are discussed accordingly. Liu et al. [1] developed a feedback-enabled wearable system for monitoring vital sign signals, especially respiration data in body sensor networks. Preejith et al. [2] developed low-power ECG hardware for un-interrupted and long-lasting control of human physiological signals, while they oversimplified to consider the energy-efficient algorithm and its hardware implementation. Jhuang et al. [3] designed tele-healthcare architecture with signal processing to help patients and physicians in detecting disease accurately, while totally ignoring the energy savings and novel chip design concepts. Shen et al. [4] proposed a wearable integrated physiological monitoring system for realizing the continuous, dynamic and long-term acquisition of human physiological signals. Von Rosenberg et al. [14] investigated the feasibility of recording the electrocardiogram (ECG), respiration, and electroencephalogram (EEG) from face-lead locations by embedding multiple electrodes within a standard helmet. They also presented a multivariate R-peak detection algorithm suitable for noisy environments. Recordings in real-world scenarios support a proof of feasibility while recording vital signs (i.e., EEG from their proposed smart helmet). Scalise et al. [15] presented a novel measurement method for detecting respiratory activity (respiration rate and respiration period) based on the use of a continuous wave (6 GHz) microwave radar reflectometry technique. Sprager et al. [16] proposed a multi-purpose technique for analyzing and evaluating different bio-signals and their impact on human health, but did not focus on power-efficient wearable devices, algorithms development, or validation of the results on the hardware. Sweeney et al. [17] designed decomposition-based methods for examining the human respiration system. Di Pascoli et al. [5] examined, electroencephalogram and electromyogram data sets by performing several experiments over the chicken embryo model, but did not focus the state-of-the art wearable devices and the power drain problems. Kesper et al. [18] examined sleep disorder patterns by adopting a 1-lead ECG channel, but their research did not concentrate on wearable device development or energy-efficiency in the healthcare environment. Mohammadi-Koushki et al. [19] proposed heart rate and respiration-related methods for managing the health of critical-condition patients, but they totally ignored the wearable device design with a power-saving concept for accurate information transmission. Zhu et al. [20] presented detailed reviews of sensors and their applicability for newborn babies, their frameworks, and other related previous research. Gargiulo et al. [21] developed an innovative and reliable scheme for managing and monitoring several pervasive medical applications, but did not consider energy-efficient wearable systems. Charlton et al. [22] presented a detailed description and comparison of several techniques related to respiratory and heart-rate monitoring activities, but they did not emphasize novel wearable systems for energy-efficient communication in healthcare. Molnar et al. [6] presented a new class of analog low-pass filters, obtained by the approximation of the rectangular magnitude by using the Bernoulli polynomials. The designed filters have equi-ripple magnitude responses in the lower part of the pass band, non-equi-ripple behavior at frequencies approaching the cutoff, and rather steep transition bands. Brugarolas et al. [7] developed joint ECG and PPG-based wearable systems, but they did not consider the energy efficiency aspect.

Dieffenderfer et al. [23] presented wearable sensor devices for medical health monitoring, but did not discuss power-efficient chip development with maximum energy savings for smart applications. Dieffenderfer et al. [24] designed a wearable sensor system consisting of a wristband and chest patch to enable the correlation of individual environmental exposure to health response for understanding the impacts of ozone on chronic asthma conditions. The wrist-worn device measures ambient ozone concentration, heart rate via plethysmography (PPG), three-axis acceleration, ambient temperature, and ambient relative humidity. Majumder et al. [25] designed textile-fabric-enabled sensor nodes for supporting medical technologies for continuous monitoring, while they did not focus on power-efficient wearable devices and algorithms and their validation over the hardware. Sodhro et al. [26] proposed a wireless body sensor network-based energy-efficient health monitoring system; they considered human vital sign signals such as ECG transmission over a real-time dynamic wireless channel. Sodhro et al. [27] developed novel energy- and battery-efficient algorithms for media streaming in WBSNs during the health monitoring of heart-attack patients; they also proposed a framework of media streaming in medical health. Hassan Sodhro et al. [28] proposed the green video transmission concept in WBSNs by adopting real-time medical video traces and analyzed the amount of energy depleted during video transmission and its impact on the lifetime of the network. Sodhro et al. [29] designed a transmission power control algorithm from the aspect of the fifth generation (5G) trends in sensor node size, and they described the significant role of the emerging technologies in healthcare applications. Sodhro et al. [30] designed a battery-efficient and sensor node lifetime-extending scheme in capsule endoscopy; they also found the impact of energy and battery charge drain on the overall network performance in patient health monitoring. Sodhro et al. [31] presented a Quality of Service (QoS) optimization algorithm in medical health; they considered the video streams and tested these traces on their method and then analyzed the entire level of QoS provided to the end users. Hassan Sodhro et al. [32] developed secure algorithms for the safe monitoring of healthcare; their algorithm is simple and effective and can be applicable for future medical networks.

Many contributors have proposed different algorithms for IoT-based wearable devices, power consumption and its impact on ubiquitous sensing, noise reduction and extracting original information signal. The usage of filters is discussed extensively in the literature, and some of the work has been presented in this paper. Siedenburg and Dorfler [33] propose audio de-noising by thresholding time-frequency. The general framework of time-frequency soft-thresholding was presented to improve denoising quality. It was also shown that simple non-iterated operators perform better compared to cutting-edge methods when evaluating signal-to-noise ratio (SNR). Agrawal et al. [34] developed different types of linear and nonlinear filters for noise reduction from corrupted images. The purpose of these filters is to provide better performance and eliminate impulse noise or Gaussian noise and remove errors from highly-corrupted images. Bhagat et al. [35] present audio filtering using extended high-pass filters. Different formulas and equations were designed for the efficient implementation of time-varying filter applications. Singh et al. [36] designed digital filters for the removal of noise signal. The time domain and frequency domain representation of the signal was performed using the fast Fourier transform technique. The Butterworth filter is used to reduce the noise from signals with different frequency and ripple factor. Singla et al. [37] present noise reduction using Butterworth, Chebyshev, and elliptical filters. The noise reduction system used depends on the application. In some cases, the requirement is to increase the overall speech quality. Adaptive filtering techniques are proposed to evaluate and examine the noise-contained signal. Students of the Artificial Intelligence Research Unit, Faculty of Engineering, Malaysia [38] propose and study methods and techniques used for noise reduction in audio applications. The best filter is the Butterworth Infinite Impulse Response (IIR) filter that uses less memory and gives a flat magnitude response. The best method applied to it is a hidden Markov model. For noise filtering, an adaptive filter is the best choice and it can be used with different methods to improve the performance of the audio signals. Students of Red Sea University [39] propose the performance of a median filter based on window sizes to remove salt and pepper noise in RGB images. Salt and pepper noise is a type of impulse noise generated when images transmit through the internet or when analog-to-digital conversion occurs. They showed the MATLAB based results by cascading the median filter at the high level of window size, because of its remarkable performance in RGB images with high and low noise densities. However, larger window size is not the suitable candidate due to its random and blurred noise effect.

A group of students from Australia and Bangladesh [8] present the performance and analysis of noise reduction through an adaptive filter using the NLMS algorithm. The system of the adaptive filter depends on the effects of step size, number of filter coefficients, number of samples, and input noise level by considering a speech signal. The parameters individually showed best performance having optimum values. IEEE members Andersen and Marc [40] propose an adaptive time-frequency analysis scheme using an asymmetric window. This technique is suitable for audio noise reduction in the low delay of 0–4 ms and has less computational complexity. The application is in real-time sound devices. The reduction in the delay is obtained by applying an Finite Impulse Response (FIR) filter to the signal as a gain and computational complexity is reduced by using the Fast Fourier transform (FFT) technique. Tahir et al. [41] design the various filters to remove the noise from the magnetic resonance imaging (MRI) of the brain due to the sensitive nature of the MRI signals.

The authors used different filters, and concluded that improving the performance of MRI images depends on the type of filtering technique used. Median filter gives a better result in the MRI brain image as compared to others. Salih proposed the effects of a low-pass filter in audio signal noise reduction. The sound sample in wave format was taken and passed through a low-pass filter and analyzed by taking different frequency and ripple factor [42]. The filter implements different equations. These equations and their function in terms of low-pass filter are explained. He concludes that the low-pass filter reduces the noise to a greater extent when it is implemented. Djurovic presented the removal of salt and pepper noise from digital images. He gives his results for applying a block matching 3D filtering (BM3D) method to the decision-based adaptive median filter [43]. His results were good and reduced noise level by up to 2 dB in both grayscale and color images. Sonia et al. [44] proposed a bilateral filter and a minimum mean square error filter for noise reduction. Their approach includes two main steps—one is the generation of a reference image from a noisy image to extract the patches, and the other is to apply a bilateral filter to each patch. This has the benefit that it does not affect the image’s original appearance. The algorithm has been tested at low, medium, and high noise density to check the performance on color images. This paper concluded that the bilateral filter was best for the removal of salt and pepper noise from images. Priyanka et al. proposed the reduction of noise in remote sensing image by using a Kalman filter, and recovered the original image from a corrupted image [45]. The Kalman filter was compared with the Wiener filter. The remote sensing image faced Gaussian noise as well as salt and pepper noise. The Kalman filter gave a better response and good efficiency in noise reduction as compared to the Wiener filter. Biswas et al. designed a technique to improve the efficiency of the Kawahara filter in reducing the noise. The Gabor Kuwahara filter was proposed, which decreases the noise without harming the edges of information [46]. Authors in References [47,48,49,50,51,52] present ECG filtering methods, but they do not focus on the energy-efficient communication in healthcare. Joao Caldeira et al. [53] propose a novel method for the medical monitoring of patients, but they do not focus on wearable device development or energy-efficient solutions. Haghi et al. [54] present the emerging role of the wearable devices in the medical market, with many challenges and remedies. Researchers in [55,56,57,58,59] designed various state-of-the-art methods for Green, secure, and healthy medical environments with cost-effective facilities.

## 3. Materials and Methods

### 3.1. Wearable Platform for ECG Collection

**Figure 1:**
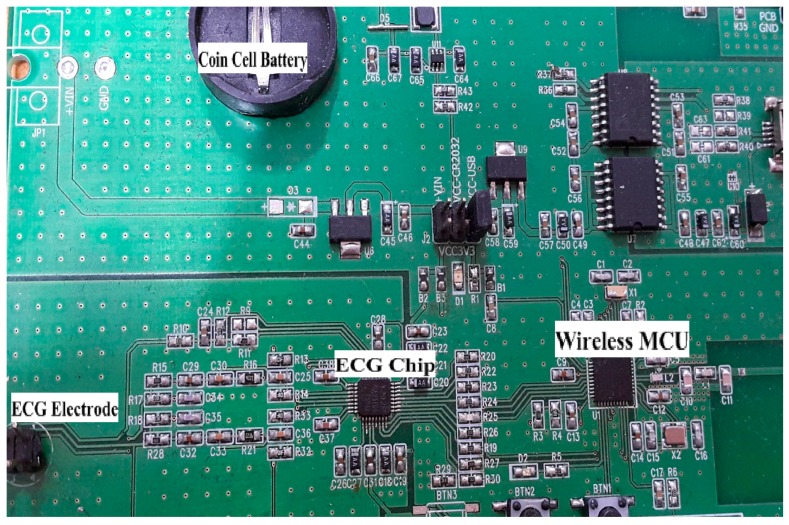
Printed circuit board (PCB) of ADS1292R with wireless micro control unit (MCU).

**Figure 2:**
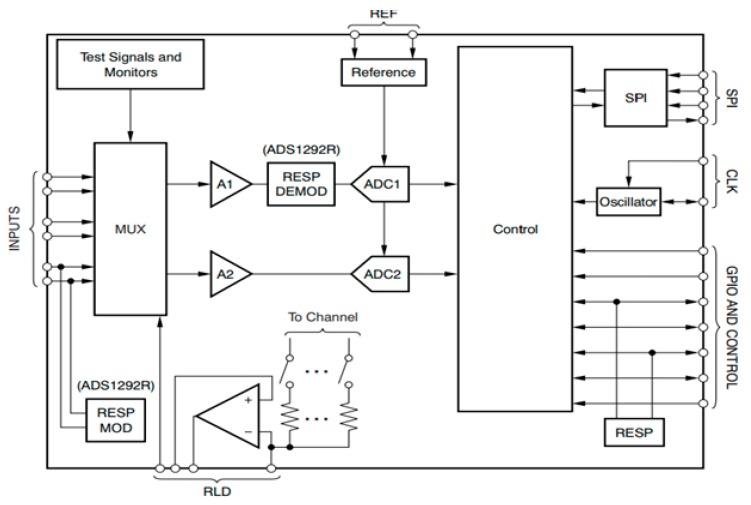
Block diagram of the ADS1292R chip. ADC: Analog to Digit Converter, RESPMOD: Respiration Modulation, RESPDEMOD: Respiration Demodulation, SPI: Serial Peripheral Interface, A1: Instrumentation Amplifier 1 gain, MUX: Multiplexer, RLD: Right Leg Drive, CLK: Clock, Gpio: General Purpose Input Output, RESP: Response.

The key ingredient in ubiquitous or pervasive sensing in sensor-enabled wearable devices is. In this study, a single chip-based wearable ECG and respiration system was designed by adopting analog front end (AFE) chip model ADS1292R, manufactured by Texas Instruments. This chip has two channels: one for real-time continuous ECG monitoring and another for real-time continuous respiration monitoring. An ADS1292R printed circuit board (PCB) with a CC2540F256 wireless micro control unit (MCU) and a physical data rate of 1 Mbps was used for Bluetooth Low Energy (BLE), as shown in Figure 1. Besides, these two channels and the AFE were built into an Right leg drive (RLD) driver circuit with lead-off detection and medical graded test signal. A block diagram of the ADS1292R chip is shown in Figure 2. The wearable platform based on the ADS1292R chip contained two 24-bit delta-sigma analog-to-digital converters (ADCs) with programmable gain amplifier with a gain value from 1 to 12. The sampling rate for the ADCs was selected from 125 samples per second (SPS) up to 8000 SPS. Digital data was controlled through Serial Peripheral Interface (SPI) communication.

**Figure 3:**
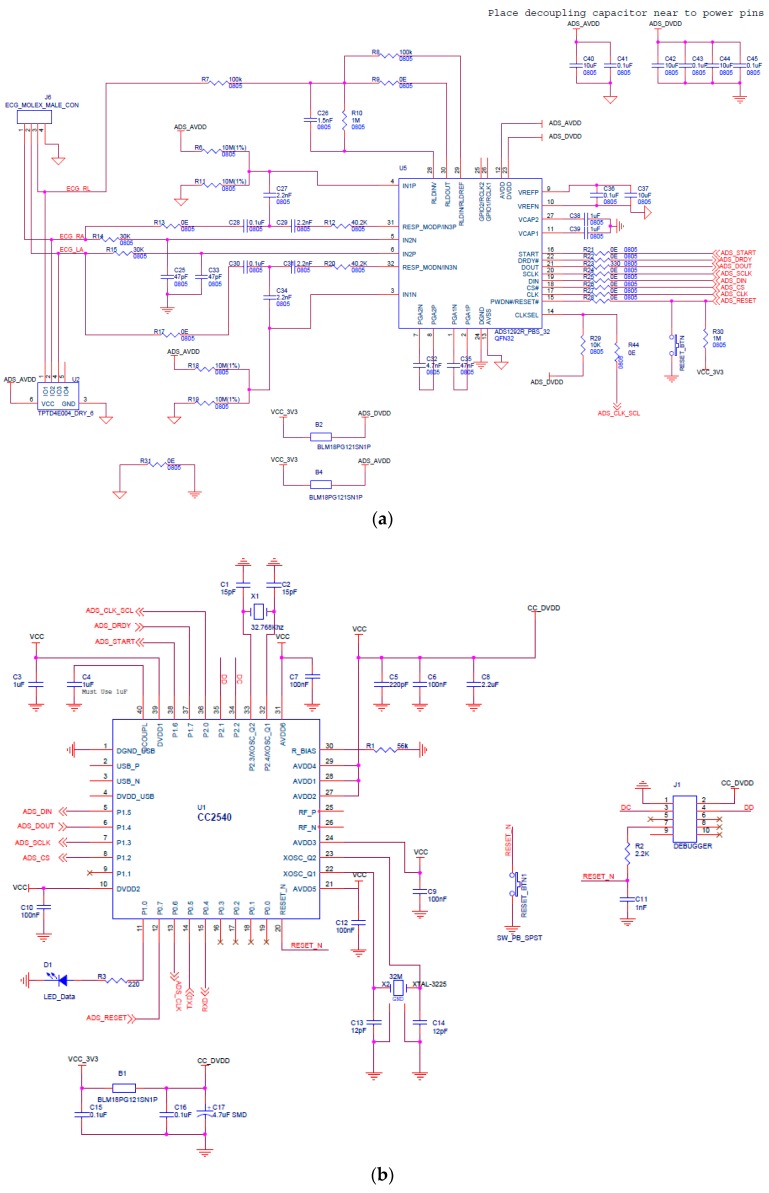
Schematic diagrams: (**a**) wearable platform with wireless MCU; (**b**) CC2540F256.

**Figure 4:**
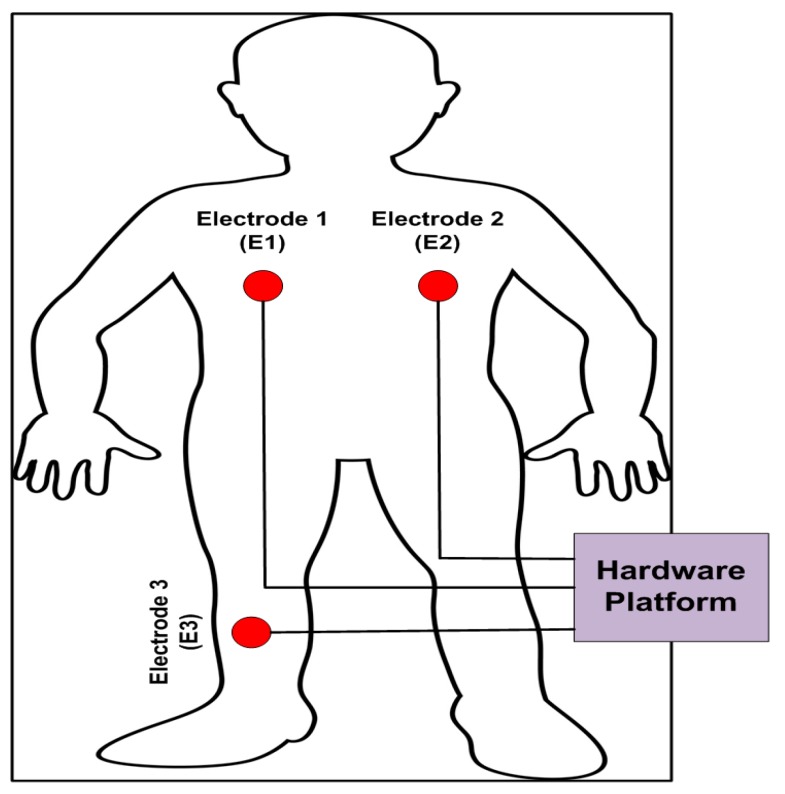
The measurement of electrocardiogram (ECG) with a single lead standard Holter.

The ADS1292R was interfaced with the wireless microcontroller thorough SPI communication. Two simultaneous channels (ECG and respiration data rate) were collected at 250 SPS through the SPI interface and then processed and transferred to PC application using Bluetooth Low Energy (BLE) technology. The schematic with the wireless MCU is shown in Figure 3. The measurement of ECG by using the developed wearable platform adopts a standard Holter with a single lead, and three inter-connected (E1–E3) points as depicted in the Figure 4.

The obtained lead was located near the cardiac axis. High-quality ECG signal is obtained by this efficient and accurate method with proper adjustment/location selection of the cardiac axis with better reputation in the clinical tasks. Besides, crystal-clear examination and diagnosis with useful information transmission can be obtained by this lead selection and cardiac axis placement technique. In this research, Ag/AgCl electrodes are considered for collecting and recording the human vital sign signals during the experimental set-up. In our test-bed, two electrodes were located on the chest surface (#l and #2) very close to the heart, while a third reference electrode (#3) was positioned on the lower-left position with a distance of 10 cm from electrode #1. This set-up offers neater and cleaner ECG signals with maximum productivity with higher R-peak amplitude and QRS-complex waves. One side was attached to the skin, while the other side was connected to the developed wearable device through the metal buttons. Other researchers [9,10,11] have also worked on hardware-based platforms for the ECG data collection through wearable devices for experimental setup. For further details of the developed ECG device, its different modes, power drain and battery type are given in the Table 1.

### 3.2. Filtering of ECG Signal

To properly remove noise from ECG signals, a notch filter (cutoff frequency 60 Hz), high-pass filter (cutoff frequency 0.67 Hz), and finally a low-pass filter (cutoff frequency 100 Hz) were designed with bilinear transformation. The filters were designed by using bilinear transformation, and the algorithm implemented in C language was Biquade Direct Form Transposed-II.

In general, the physiological signals captured from the human body using wearable devices have some additional unwanted noise. Therefore, it is crucial to eliminate that undesired information from bio-signals so that clear signals can be obtained for further utilization in healthcare applications. Various kinds of noise can affect the strength of bio-signals collected from the human body; the hardware platform cannot completely filter all these noises. Hence, it is paramount to apply appropriate filters to exclude unwanted information from the originally captured signals from wearable devices. Since hardware filters depend on capacitors (which are considered as the primary restraint for entirely removing noisy information), their justification is not well addressed from both the effective construction and high visibility point of view.

**Figure 5:**
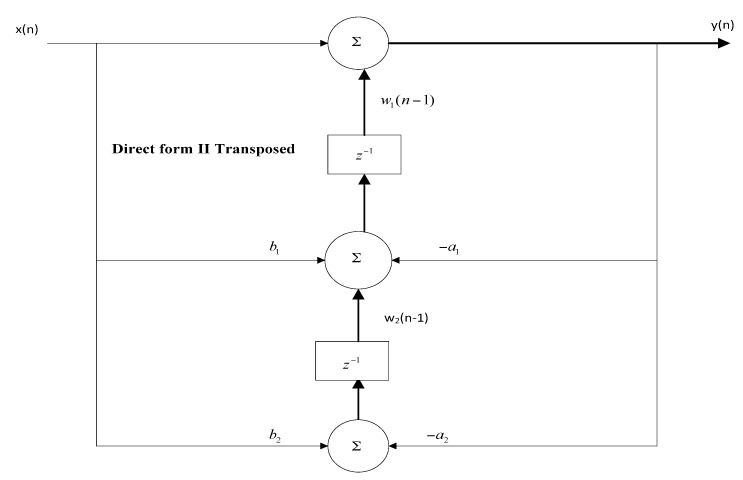
Block diagram of Biquade Direct Form Transposed-II.

Subsequently, software filtering is commonly reliant on cut-off frequencies which can be precisely controlled by consenting implementation of innovative filter models. The signal levels are enormously small (i.e., 1 mV for bio-signals such as ECG), and it is crucial to apply filtering to eradicate a wide range of unwanted noisy signals [8,37,38,39,40]. The noise in ECG signal is mainly due to unstable DC offset between the electrode–human body interaction, electrical instrumental noise in the environment, power-line (50/60 Hz), muscle noise, and internal noise while manufacturing wearable ECG devices [41]. In this section, we found that due to the sensitive nature of heart rate data, it is very necessary to remove the power line noise before sending to the computer interfaced with the end user. The second-order bi-quad notch filter was designed and applied to the real-time ECG data by using bilinear transformation from analog to digital filtering. The filter coefficients for the notch filter were calculated by the relationship in Equations (1)–(7) and Figure 5.

X(s) and Y(s) are the input and output, respectively, while $X_{i}$ and $X_{j}$ are the samples in taps i and j at time s, which can also be considered for the Y(s). $A_{i}$ and $B_{j}$ are the parameters of the filter (which can be different for each tap), and the Ki are a set of internal variables.

(1) K=tan (π×w)

whereby w is a normalized cut-off frequency with constant pi (π) value of 3.141592653.

(2) $$\begin{array}{c} norm=1/(1+K/Q+K\times K),\\ a_{0}=(1+K\times K)\times norm,\\ a_{1}=2\times (K\times K-1)\times norm, \end{array}$$

where $a_{2}$ and $b_{1}$ are the filter coefficients computed during the filter’s runtime. $b_{0}$ and $b_{2}$ multiply the input signal X(s) and are referred to as the feedforward coefficients; similarly, $a_{1}$ and $a_{2}$ multiply the output signal Y(s) and are known as the feedback coefficients.

(3) $$b_{2}=\left(1-K/Q+K\times K\right)\times norm$$

In the above equation, Q is a quality factor with a value of 0.707. The algorithm implemented in the MCU, is transposed two times in a linear fashion, as shown in Figure 5.

(4) $$\frac{Y(z)}{X(z)}=\frac{b_{0}+b_{1}z^{-1}+b_{2}z^{-2}}{1+a_{1}z^{-1}+a_{2}z^{-2}}$$

(5) $$Y(n)=b_{0}x(n)+w_{1}(n-1)$$

(6) $$w_{1}(n)=b_{1}x(n)-a_{1}y(n)+w_{2}(n-1)$$

(7) $$w_{2}(n)=b_{2}x(n)-a_{2}y(n)$$

## 4. Proposed Energy-Efficient Algorithm

We propose an energy-efficient transmission power control (ETPC) algorithm and validate its performance during ECG data transmission over the joint hardware and software platform. Transmission power (TP) is adjusted according to the variations in the dynamic wireless channel and desired demand from the base station (BS). ETPC is proposed by modifying the adaptive power control algorithm in [26], but the power allocation strategy is different in both, and ETPC is applicable for both static and dynamic scenarios, unlike Adaptive Transmisison Power Control (ATPC) (only for dynamic case) in [26]. Besides, the proposed ETPC is compared with the orthodox TPC, such as Gao’s, constant TPC, and Xiao’s methods [26], which do not properly follow the characteristics which leads to a sacrifice of either the efficiency savings or the channel reliability; for further detail, see [26]. The conventional methods do not consider all aspects of the channel while designing the power control algorithms, and due to less synchronization between the demands of the application and their solutions, most of the time power is wasted in control packets and sending feedback and acknowledge (ACK) information (i.e., Gao’s and Xiao’s methods). In constant TPC, direct high power is provided in a linear fashion, which compromises either energy or reliability, which is not applicable in practice. The main experimental parameters are lowest received signal strength indicator (*RSSI*) sample, $R_{lowest}$, which is preferred after the loss/drop of the latest RSSI sample in the start of first transmission; RSSI average, $\bar{R}$, is the estimated average of RSSI samples; RSSI target, $R_{target}$ (−85 dBm)—its value lies between the constant lower threshold (TRL) and variable higher threshold ($TRH_{\mathrm{var}}$ with value −83 dBm); averaging weight $\alpha_{1}$ of good channel (i.e., with high RSSI and less packet drop); and averaging weight $\alpha_{2}$ of bad channel (i.e., with less RSSI and slightly high packet drop). ETPC uses an adaptive on-demand mechanism to find and fulfill the best transmission power level and requirement of the user, respectively. More details can be found in [26].

(8) $$\bar{R}=R_{lowest}+(1-\alpha_{1})\times \bar{R}$$

(9) $$\bar{R}=R_{lowest}+(1-\alpha_{2})\times \bar{R}$$

(10) $$\Delta P=\{\begin{array}{c} 2\\ -1\\ 0 \end{array}\begin{array}{c} if\\ if\\ if \end{array}\begin{array}{c} \bar{R}<TRL\\ \bar{R}>TRH_{\mathrm{var}}\\ TRL<\bar{R}<TRH_{\mathrm{var}} \end{array}$$

where ΔP shows the change in transmission power level, and is adapted according to the variation in the wireless channel and received signal strength indicator (RSSI) requirement.

(11) $$TRH_{\mathrm{var}}=TRL+\sigma$$

(12) $$\sigma =\sqrt{\frac{1}{n}\sum_{i=1}^{n}\left(R_{i}-\bar{R}\right)},\;i=1,2,\ldots ,n$$

where σ and n are the standard deviation in dBm and number of RSSI samples, respectively.

**Figure 6:**
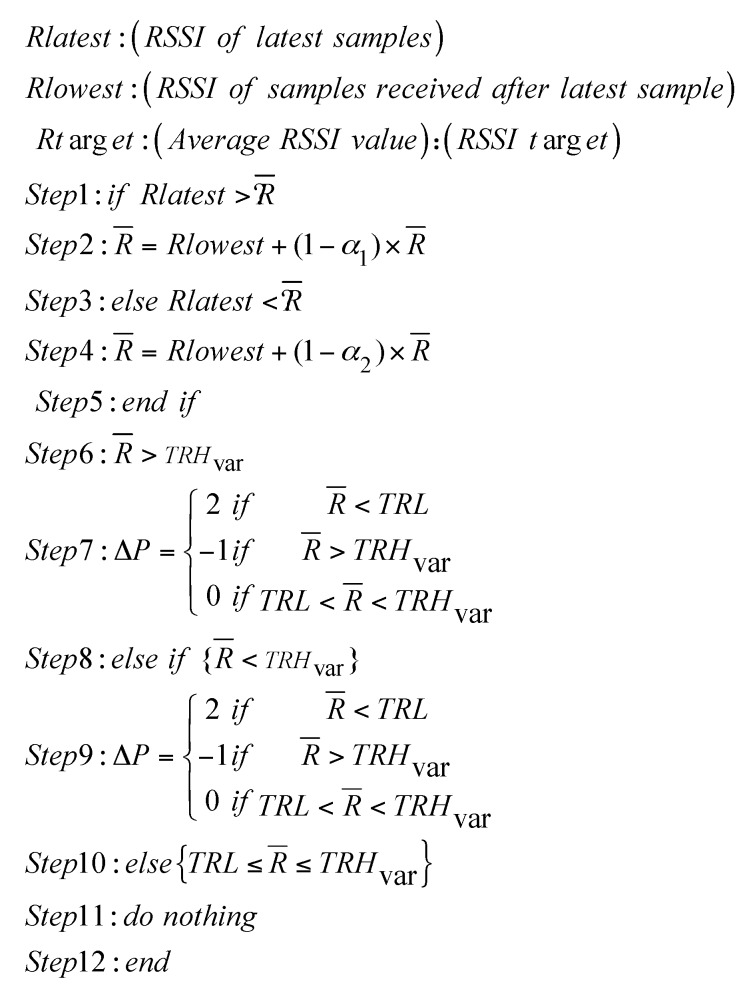
Pseudo code of the proposed transmission power control (TPC)-based energy-efficient (ETPC) algorithm. *RSSI*: received signal strength indicator; $\bar{R}$: average *RSSI*; *TRH*_var_: variable higher threshold; *TRL*: constant lower threshold.

**Figure 7:**
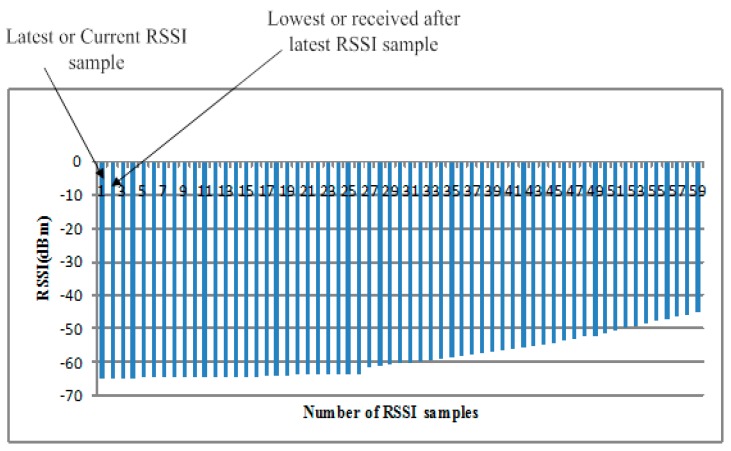
RSSI data samples during transmission.

Fair allocation of transmission power offers more stable and long-lasting communication. Lowest RSSI samples $R_{lowest}$ gives un-interrupted transmission between transmitter and BS nodes (which is why it is one of the main ingredients), and its loss heavily impacts the transmission pace and creates discontinuity in data transmission. Traditional constant TPC, and other methods such as Gao’s and Xiao’s consider fixed RSSI threshold values, not properly looking at the dynamic nature of the wireless channel. We assume that the proposed ETPC is monitored by both BS and transmitter node with uplink data transmission in a linear on-demand fashion. BS decides and allocates the next TP level by calculating the average RSSI ($\bar{R}$) of all data samples. We assumed that the transmitter node had the record of each RSSI data sample, and by considering that information, power level was assigned accordingly. Transmission power and lowest RSSI samples are represented as $P_{t}$, $R_{lowest}=R_{latest}-1$, respectively, as shown in Figure 6 and Figure 7. The BS monitors and computes $\bar{R}$ from occasionally by using Equations (8) and (9) to examine and identify the channel states accordingly. ETPC consumed less resources and gave the higher energy-efficiency of 35.5%, and is applicable for both static (i.e., sitting and standing) as well as dynamic (i.e., walking and running) body postures, unlike the conventional methods [26]. The main limitation of the proposed ETPC is the compromise of the channel reliability (i.e., greater packet loss ratio, PLR), which will be minimized in the near future. Interested readers are referred to [26] for further detail of the conventional methods.

## 5. Experimental Results and Discussion

**Figure 8:**
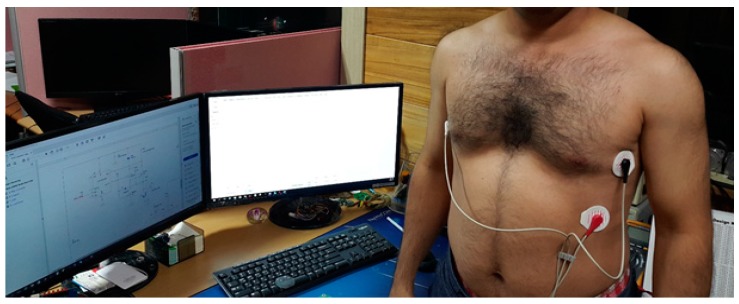
Experimental hardware set-up for wearable ECG signal collection.

**Figure 9:**
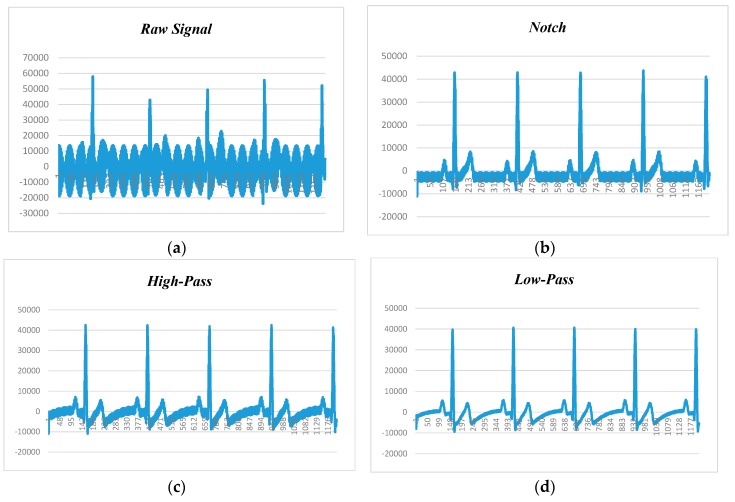
ECG data filtering at 60 BPM and 60 Hz noise: (**a**) raw data; (**b**) notch filter; (**c**) high-pass filter (HPF); (**d**) low-pass filter (LPF).

**Figure 10:**
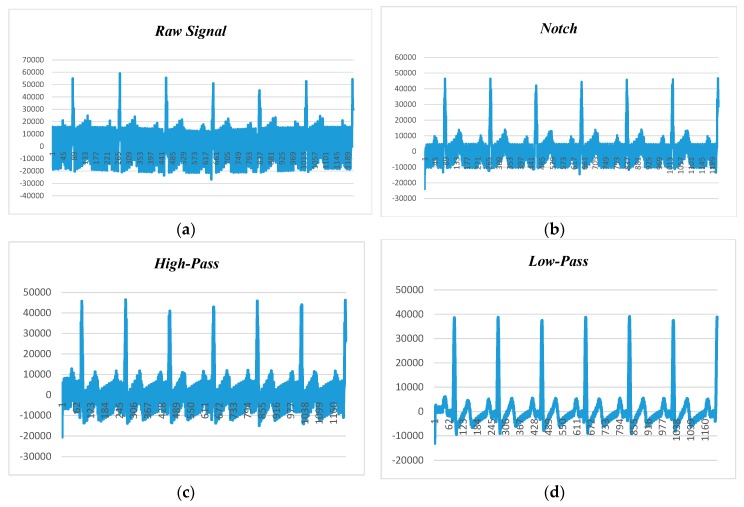
ECG data filtering at 80 BPM and 50 Hz noise: (**a**) raw data; (**b**) notch filter; (**c**) high-pass filter (HPF); (**d**) low-pass filter (LPF).

**Figure 11:**
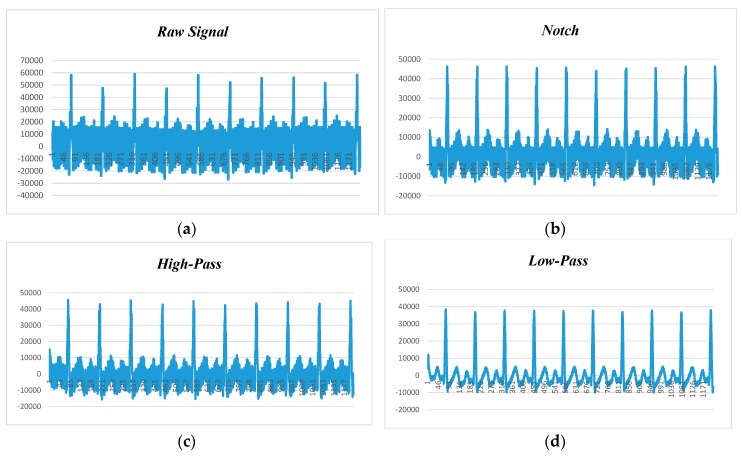
ECG data filtering at 120 BPM and 50 Hz noise: (**a**) raw data; (**b**) notch filter; (**c**) high-pass filter (HPF); (**d**) low-pass filter (LPF).

**Figure 12:**
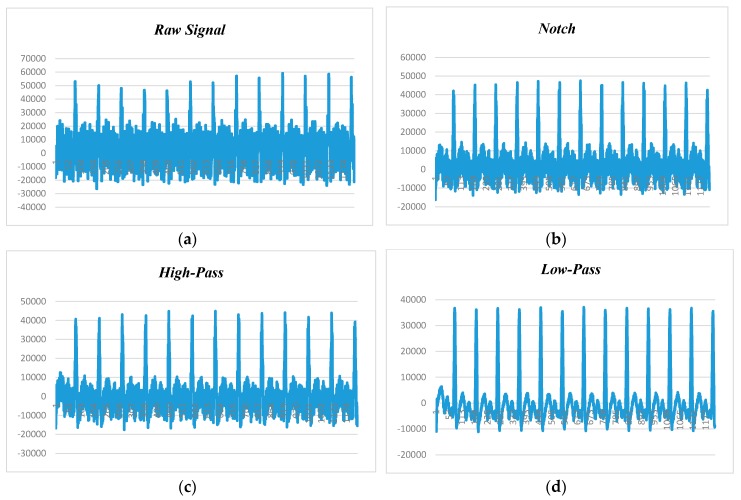
ECG data filtering at 160 BPM and 50 Hz noise: (**a**) raw data; (**b**) notch filter; (**c**) high-pass filter (HPF); (**d**) low-pass filter (LPF).

**Figure 13:**
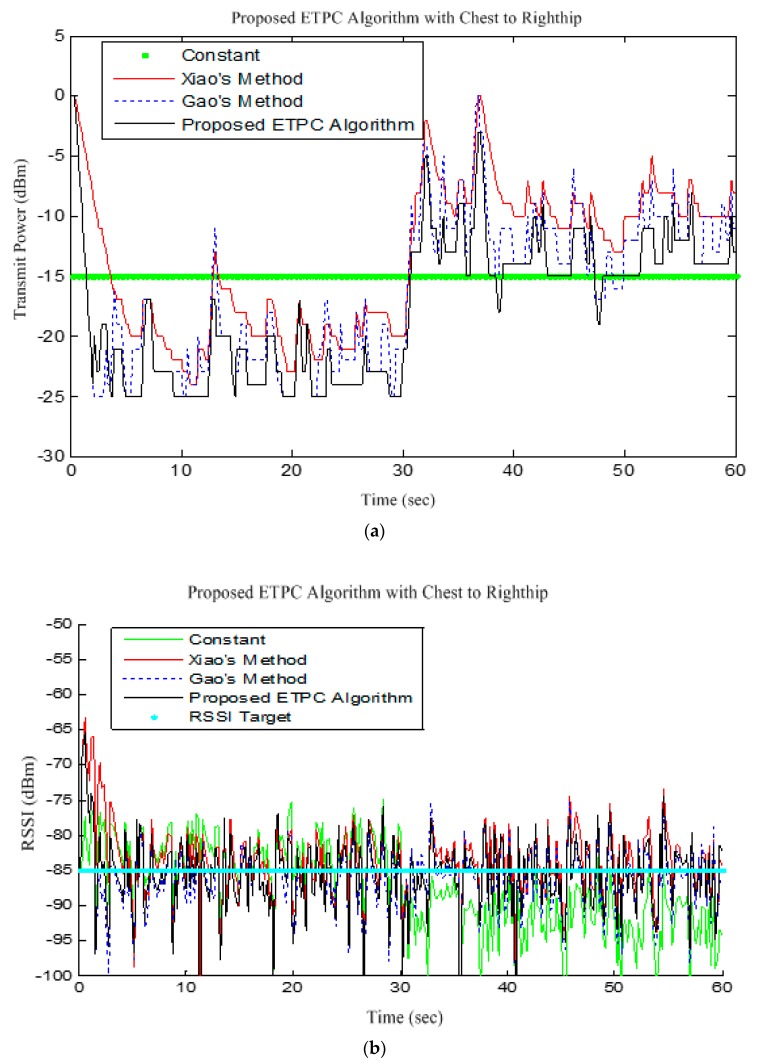
Transmission power level and RSSI in chest to Righthip: (**a**) Transmission power level; (**b**) RSSI samples of each packet.

Our experiment had 30 subjects with no history of cardiovascular disease as participants. The subjects were tested under the standard time period between 9:00 a.m. and 4:00 p.m., at a room temperature of 21–26 °C. In this section, we analyze the performance of single-chip-based wearable platform for measuring ECG signal by applying different filters for power line noise filtering. The performance of the three types of filters was evaluated while rectifying the ECG signal. Moreover, the traditional transmission power control (e.g., constant TPC, Gao’s and Xiao’s algorithms) were compared with the proposed ETPC through a vast experimental set-up over a hardware- and software-integrated platform by considering aggregated RSSI and transmission power values. Experiments for generating human body heart rate were performed at rest and on a bike. When a participant uses a bicycle their heart rate increases, and when the bike is not used, then the pedaling heart rate gradually comes to rest. Human ECG data was used to validate the heart rate algorithm, and has the capability to generate different noise—for example, AC power line noise, cabling noise, and baseline wandering. For our experimental setup, the ECG signal was coming from the human body during bike riding/driving. An ECG simulator was used to validate the signal and to generate different kinds of noise (e.g., power line noise at 50 Hz and 60 Hz, miscellaneous noise that almost corrupts ECG signal, and baseline wandering noise for algorithm validation). The first prototype ECG signal was used on a personal computer (PC), then the ECG device wirelessly transmitted raw data that was captured by a Bluetooth Low Energy (BLE) dongle device connected to the PC. A PC application was developed that displays ECG signal and does the filtering and ECG processing (e.g., calculation of heartbeat from raw ECG data). Moreover, the adopted ECG simulator is from Fluke “Fluke Biomedical 215A Patient Simulator”, which generates different signal artifacts (e.g., power line noise of 50 Hz or 60 Hz, baseline wandering, miscellaneous noise, etc.). Our developed device prototype had a size of 6 cm by 12 cm, but the final product will have a size of 4 cm by 4 cm, and we used general electrodes rather than medical electrodes. We used real-time ECG data sets from our developed wearable ECG device by adopting the experimental scenario, chest to righthip. Notch, high-pass, and low-pass filters were deployed on the hardware to remove the power-line noise and artifacts from the generated real-time ECG data, and were then compared. Average transmit power was used to analyze the energy-efficiency of our proposed ETPC algorithm in comparison with conventional TPC methods, and results showed that the proposed ETPC algorithm enhanced energy efficiency by up to 35.5%. Figure 1, Figure 2, Figure 3, Figure 4, Figure 5, Figure 6, Figure 7 and Figure 8 present the hardware set-up, filter design, and TPC algorithms implementation over the test-bed. Additionally, ECG filtering with several different filters is discussed and depicted by comparing their noise removal performance in Figure 8, Figure 9, Figure 10, Figure 11 and Figure 12. Figure 13 shows the comparison of transmission power (dBm) and corresponding received signal strength indicator (RSSI) values in the first 60 s between conventional TPC methods and proposed ETPC with the experimental scenario chest to righthip in frequency band 2.4 GHz. In addition, it was observed and examined that the constant TPC method could not adjust dynamic channel states and performed poorly by consuming more energy in the case of good channel state or sacrificed reliability in the case of bad channel condition.

Figure 9, depicts the human ECG signal at 60 beats per minute (BPM) and 60 Hz noise level, in which Figure 9a–d show raw ECG signal and filtered signals by notch, high-pass, and low-pass filters, respectively. Since BPM was 60, each peak should appear in 1 second. The first peak recorded is at 161 and second is at 410. Calculating the difference yields 250. Our sampling rate was 250 samples per second (SPS), so multiplying 1 by 250, we will get 250 SPS. After applying the notch filter, the first peak is at 157 and the second peak is at 412, so the difference is 255, and the sampling rate is 255 SPS.

Figure 10 presents the human ECG data at 80 beats per minute and 50 Hz noise level. Figure 10a shows the raw ECG data before applying the filtering techniques, while Figure 10b–d depict the filtered data with adaptation of notch, high-pass, and low-pass filters. It is found that the low-pass filter performed better than the notch and high-pass filters, and the high-pass filter had better noise filtering capability than the notch filter. Hence, it can be claimed that the low-pass filter is the suitable candidate for power-line noise and artifact removal in real-time heart rate variability data (i.e., ECG).

Figure 11 displays the human ECG data at 120 beats per minute and 50 Hz noise level. Figure 11a shows the raw data before applying filtering techniques, while Figure 11b–d depict the filtered data with the adaptation of notch, high-pass, and low-pass filters. We observed that the low-pass filter performed better than the notch and high-pass filters, and the high-pass filter had better noise filtering capability than the notch filter. Hence, it can be claimed that the low-pass filter is the suitable candidate for power-line noise and artifact removal in real-time heart rate variability data (i.e., ECG).

Figure 12 presents the human ECG data at 180 beats per minute and 50 Hz noise level. Figure 12a shows the raw data before applying filtering techniques, while Figure 12b–d depict the filtered data with the adaptation of notch, high-pass, and low-pass filters. We found that the low-pass filter performed better than the notch and high-pass filters, and the high-pass filter had better noise filtering capability than the notch filter. Hence, it can be claimed that the low-pass filter is the suitable candidate for power-line noise and artifact removal in real-time heart rate variability data (i.e., ECG).

In summary, the experimental results were drawn by adopting 80 BPM, 120 BPM, and 160 BPM (all at 50 Hz noise in Figure 9, Figure 10, Figure 11 and Figure 12) for noise reduction in real-time ECG data with notch, high-pass, and low-pass filters, respectively. It was observed and examined that ECG signal deteriorated more at the higher value of BPM, and less at the lower BPM. The low-pass filter outperformed the other filters, and the high-pass filter performed better than the notch filter in minimizing power-line noise level and artifacts, as depicted in Figure 8, Figure 9, Figure 10 and Figure 11. Figure 12 reveals the transmission power and received signal strength indicator (RSSI) performance for the proposed ETPC algorithm and typical conventional methods.

Figure 13 illustrates the transmission power (TP) and respective RSSI value for the ETPC algorithm and typical TPC methods with “Chest to Righthip” scenario, testing on the hardware platform. From the experimental results, it is clear that the proposed ETPC algorithm transmits at low transmission power (as shown in Figure 13a) and relatively stable RSSI value (i.e., less deviation, 5.43 dBm, and higher packet loss ratio, 5.51%) as given in Figure 13b, while the constant TPC method consumes more transmit power, has stable RSSI value (i.e., deviation of 7.53 dBm) and less packet loss ratio (2.87%) than Gao’s (deviation of 5.57 dBm) and Xiao’s (deviation of 5.76 dBm) methods, as presented in Figure 13a,b demonstrates that our proposed algorithm exploits a smaller RSSI of −88 dBm as compared to conventional TPC methods. This means that the proposed algorithm (3.75% packet loss) had slightly less reliability than Gao’s (3.69% packet loss), Xiao’s (3.53% packet loss), and constant TPC (2.87% packet loss) methods in the “Chest to Righthip” experimental scenario. Generally, there was less variation in “Chest to Righthip” with the proposed ETPC algorithm and conventional TPC methods. Our proposed algorithm exhibited less TP drain (or more energy saving), high RSSI stability, and greater packet loss ratio than Gao’s, Xiao’s, and constant TPC methods—in other words, the proposed ETPC surpassed the typical conventional TPC methods.

## 6. Conclusions and Future Work

Ubiquitous or pervasive sensing is the center of everyone’s attention while playing with wearable devices for smart healthcare. However, the main energy consumption problem always keeps them one step back from their potential. This paper first develops a single-chip-based wearable wireless electrocardiogram (ECG) monitoring system by adopting the analog front end (AFE) chip model ADS1292R for medical health applications. Second, filters are very important to getting noise-free and efficient diagnosis of patients’ health, so three filter types (i.e., notch, low-pass, and high-pass) were designed to rectify the power-line noise and artifacts. Third, the ETPC algorithm is proposed for the efficient transmission of the filtered real-time heart-rate activity data. Besides, the filters and proposed ETPC were implemented and validated with human ECG data (60–120 BPM and 60 Hz noise on the hardware). Experimental results reveal that our developed chip collects real-time ECG data in a more accurate and efficient way, and the proposed ETPC algorithm achieves a higher energy savings of 35.5% with little compromise in channel reliability (as indicated by packet loss ratio) compared to conventional TPC (e.g., constant TPC and Gao’s and Xiao’s methods). In the near future, Kalman and other adaptive filters will be developed for noise removal in healthcare applications. Moreover, we will apply ECG signal to develop biometric security techniques for real-time telemedicine systems.

## Figures and Tables

**Table 1:** Power consumption of different modes.

Mode	Power Consumption	Battery
Standby	160 μW overall consumption, and Low Power: 335 μW/channel	Coin cell battery
Acquisition of Noise ECG	42 mW	Ten-year battery lifetime in the absence of system power
Lead-off detected	Lowest 14.4 nW, Highest 52.8 µW	80% power is saved as compared to the conventional batteries
